# The Effect of Developmental Characteristics of Adolescents’ Perceived Social Support on Social–Emotional Competence from a Cumulative Ecological Resources Theory Perspective

**DOI:** 10.3390/bs15070921

**Published:** 2025-07-08

**Authors:** Chao Ma, Chanjuan Zhang, Wenyin Zhao, Haibo Yu

**Affiliations:** 1 Normal School, Shihezi University, Shihezi 832000, China; zhangchanjuan@stu.shzu.edu.cn (C.Z.); zhaowenyin@stu.shzu.edu.cn (W.Z.); 20212001001@stu.shzu.edu.cn (H.Y.); 2 Center for Applied Psychological Research, Shihezi University, Shihezi 832000, China

**Keywords:** cumulative ecological resources theory, adolescents, perceived social support, social–emotional competence, latent profile analysis, latent transition analysis

## Abstract

Cumulative Ecological Resources Theory offers an integrative perspective for social–emotional interventions by overcoming the traditional dichotomy between internal and external resources. As a crucial ecological resource, perceived social support is known to be heterogeneous, yet its mechanism of influence on social–emotional competence remains to be clarified. This study investigates the effect of developmental characteristics of adolescents’ perceived social support on social–emotional competence. A six-month longitudinal study tracked 995 adolescents using the Multidimensional Scale of Perceived Social Support and the Delaware Social and Emotional Competency Scale. Based on the results, (1) the adolescents’ perceived social support could be categorized into four types: Poor, Moderate, Rich, and Separated; (2) the Poor type exhibited greater category mobility, whereas the Moderate and Rich types demonstrated higher stability; some adolescents in the Poor, Moderate, and Rich types transitioned to the Separated type; and adolescents in the Separated type were more likely to transition to the Moderate type; (3) gender, age, and boarding status influenced the transition in perceived social support categories; (4) the transition pattern of transitioning to or remaining within the Rich type positively predicted social–emotional competence at T2. The findings support the Cumulative Ecological Resource Theory by revealing heterogeneity in adolescents’ perceived social support and demonstrating that trajectories toward higher resource accumulation significantly enhance social–emotional competence.

## 1. Introduction

Social–emotional competence (SEC) constitutes a core component of social adaptability, reflecting an individual’s self-awareness in social contexts and their perceptiveness in interpersonal relationships (J. Zhang & Liu, 2021). This concept’s roots trace back to early “social intelligence,” defined as an individual’s general capacity to comprehend social affairs (Sumin & Junya, 2024). Gardner later expanded this concept into the emotional domain, emphasizing affective experiences and subjective agency in social interactions (R. Guo, 2022). The Collaborative for Academic, Social, and Emotional Learning (CASEL) integrated both social and emotional dimensions to develop its framework. This framework not only encompasses the five core internal competencies—self-awareness, self-management, social awareness, peer relationships, and responsible decision-making—but also identifies the external support resources constructed through school-family-community collaboration as a key mechanism for social–emotional competence development. Therefore, it is evident that the synergistic interaction between internal and external resources occupies a critical position in this framework, and their effective integration is vital for optimizing an individual’s social–emotional competence (Collaborative for Academic, 2022). However, most CASEL–based interventions fail to adequately integrate individuals’ internal and external support resources. Thus, how to enhance individuals’ social–emotional competence through an integrated internal-external resources approach—a Cumulative Ecological Resources Theory Perspective—has become a key research focus in education.

### 1.1. Cumulative Ecological Resources Theory Perspective

The developmental resources perspective posits that cultivating adolescents’ social–emotional competence requires both internal and external resources. Internal resources refer to intrinsic qualities that facilitate effective coping when faced with challenges, such as psychological resilience. External resources encompass positive developmental experiences derived from environmental supports, including parental guidance and teacher mentorship (Dimitrova & Wiium, 2021). Current research has also explored internal resources (emotional experience, Big Five personality, etc.) and external resources (parenting style, teacher–student relationships, peer atmosphere, etc.) separately in social–emotional development (J. Li, 2023; Ma, 2024; J. Yang et al., 2023). Although this siloed research perspective facilitates the analysis of specific resource mechanisms, it tends to overlook the holistic effects of internal–external resource interactions on social–emotional competence development. The Cumulative Ecological Resources Theory transcends the traditional binary division of internal and external resources by proposing the integrated concept of “Ecological Resources.” The theory posits that adolescents’ authentic ecological environment features an interdependence of internal and external resources, wherein they acquire ecological resources through dynamic interactions with multiple socialization systems encompassing family, school, and neighborhood contexts. The basic theoretical assumption is that individuals’ positive development is linked to the cumulative experience of ecological resources (Dejenie et al., 2024). Empirical studies have shown that cumulative ecological resources positively influence adolescents’ social adaptation by alleviating depressive symptoms, reducing suicide and self-harm rates, and enhancing core values and social–emotional competence (Baza-Arce et al., 2024; Hou et al., 2022; Xiao et al., 2024; Y. Zhao et al., 2024). However, as the most critical integrated ecological resource connecting both internal and external domains, perceived social support (PSS) and its specific mechanisms in promoting adolescents’ social–emotional competence remain underexplored in both domestic and international research.

### 1.2. The Impact of Perceived Social Support on Social–Emotional Competence

Perceived Social Support encompasses both internal psychological capital, such as subjective well-being, and a wide range of external interpersonal resources. Through actively perceiving and evaluating these resources, individuals cultivate positive emotional experiences, which include three dimensions: perceived family support, perceived peer support, and perceived teacher support (Cartwright et al., 2022). From the perspective of Ecological Resource Conservation Theory, adolescents’ perceived social support resources activate both the motivational efficacy and functional efficacy of these resources; this mechanism enables adolescents to demonstrate more proactive resource utilization across diverse social contexts, thereby effectively fostering the development of non-cognitive abilities such as social–emotional competence. A prior study on South Korean early adolescents further demonstrated that tri-dimensional perceived support resources—from family, peers, and teachers—constitute critical elements for social–emotional competence development (Suryanarayana, 2022). It was previously shown that an adolescent’s perception of a warm parenting style in their family has a positive effect on the development of social–emotional competence (J. L. Song & Zhong, 2024). Teachers’ autonomous learning instructional support positively correlates with adolescents’ social–emotional competence development (Collie, 2021). Likewise, friendships directly and positively contribute to the development of social–emotional competence (Yi, 2022).

The Ecological Resources Accumulation Model further posits that the cumulative effect of cross-contextual resources promotes positive individual development more effectively than single resources (B. B. Wang, 2021). Specifically, perceived support resources from family, peers, and teachers not only function independently but also exhibit cumulative effects. Such resource accumulation is more likely to facilitate positive psychological development among adolescents. Indeed, prior research has also demonstrated that individuals belonging to high-resource environmental configurations tend to exhibit stronger social–emotional competence (Sun et al., 2023). Therefore, from a horizontal perspective, adolescents’ level of cross-contextual resource accumulation in perceived social support is closely related to the development of their social–emotional competence. However, prior research has failed to clearly articulate how cross-contextual resources are specifically “combined” at the individual level. That is, past studies have predominantly focused on the “quantitative” accumulation of adolescents’ perceived social support, while neglecting potential “qualitative” differences. Moreover, from a longitudinal standpoint, the developmental dynamics of adolescents’ social support profiles and their long-term impact on social–emotional competence remain a key area for investigation. Therefore, this study aims to answer the following Research Questions (RQs):

RQ1: Do distinct qualitative profiles of adolescents’ perceived social support exist?

RQ2: How do the different profiles of adolescents’ perceived social support develop over time?

RQ3: Does a positive transition into a “high-resource support” profile predict levels of social–emotional competence in the next measurement wave?

### 1.3. Heterogeneity in Perceived Social Support

Under the traditional dualistic perspective of internal–external resource division, research on adolescents’ perceived social support has predominantly adopted a Variable-centered Approach (J. J. Chen, 2022). This approach assumes population homogeneity and uniform variable relationships, often neglecting the heterogeneity in psychological mechanisms among adolescents with different patterns of perceived social support. Under the Cumulative Ecological Resources Theory Perspective, such disparities are particularly evident in their differential perceptions of support from three key sources: family, peers, and teachers. Based on the differences in perceptual abilities within the adolescent group regarding ecological resources, as well as the diversity and complexity of ecological resources in their respective environments, the social support perceived by individuals often exhibits heterogeneity in real life (M. L. Guo et al., 2021). Accordingly, based on this evidence and to specifically answer RQ1, we propose Hypothesis 1:

**H1:**  *Adolescents’ perceived social support can be categorized into at least three distinct profiles: High, Moderate, and Low support.*

Adolescents’ perceived social support demonstrates distinct categories. Furthermore, these perceived social support categories exhibit dynamic developmental changes, grounded in Developmental Contextualism Theory, which posits that individual–environment interactions result in non-deterministic developmental trajectories characterized by contingency and nonlinear progression (Lerner, 2021). Empirical evidence from a longitudinal study of Chinese vocational students confirms type-specific score fluctuations in perceived social support across follow-up assessments (Liu et al., 2024). Therefore, to address RQ2, we propose Hypothesis 2:

**H2:**  *Transition patterns between categories of adolescents’ perceived social support follow at least two distinct pathways: one maintaining initial category membership (demonstrating temporal stability), the other transitioning between categories (exhibiting temporal variability).*

Individual-centered Analysis Approaches, such as Latent Profile Analysis (LPA) and Latent Transition Analysis (LTA), can identify the heterogeneity in individual psychological or behavioral development by determining latent classes. Specifically, the primary objective of LPA is to identify underlying subgroups from a dataset, classifying a sample with significant heterogeneity into two or more distinct categories, each with similar characteristics. In this study, LPA allows for the simultaneous inclusion of multiple dimensions of adolescents’ perceived social support, thereby identifying different categories of perceived social support. LTA, on the other hand, describes how these categories change over time from a probabilistic perspective, utilizing a latent transition probability matrix. A transition is defined as the pattern of change in an individual’s latent class membership across different time points (Z. Wen et al., 2023). In the context of this study, a latent transition refers to the change in the categories of perceived social support to which an adolescent belongs at different time points. Previous studies using LPA revealed that individuals in the same environment can be categorized into three levels of perceived support resource availability: high, medium, and low (Petersen et al., 2023). Moreover, some scholars employed LTA to examine the developmental trajectories of perceived family support during late adolescence. The results revealed that adolescents with increasing levels of perceived family support were more likely to transition toward higher levels of social–emotional competence (Kim & Kim, 2022). In contrast, research on the potential profiles of adolescents’ perceived social support and the impact of profile development characteristics on social–emotional competence remains scarce in China. Therefore, to address RQ3, drawing upon both the nonlinear dynamics of Developmental Contextualism Theory and empirical evidence on the promotive effects of positive transitions, we propose Hypothesis 3:

**H3:**  *Adolescents’ category transition patterns toward the “high-resource accumulation type” will significantly and positively predict developmental levels of social* *–emotional competence in the next measurement wave.*

### 1.4. Predictors of Transitions in Perceived Social Support Categories

There are also a number of factors that can predict the development of adolescents’ perceived social support. As an individual’s access to social resources increases with age, the level of perceived social support increases (Ding & Liu, 2023). Additionally, the roles of gender and boarding status in the developmental trajectory of perceived social support remain inconclusive. Empirical evidence indicates that boarding adolescents demonstrate significantly lower levels of perceived social support than their non-boarding counterparts (Zou et al., 2021), whereas other studies have found no statistically significant differences between the two (W. Wen et al., 2023). Different studies have also reached inconsistent conclusions about the role of gender in the development of perceived social support (Lian, 2022). Therefore, it is necessary to explore the predictive role of the above factors on shifts in the type of perceived social support.

In summary, based on the Theory Perspective of Cumulative Ecological Resources, this study aims to examine the effect of developmental characteristics of adolescents’ perceived social support on social–emotional competence. The specific research objectives are:

(1) To identify the latent profiles of adolescents’ perceived social support.
(2) To examine the transition pathways among the latent profiles of perceived social support and to investigate the predictive roles of gender, age, and boarding status on these transitions.
(3) To explore the impact of adolescents’ perceived social support profile transition patterns on social–emotional competence.

## 2. Methods

### 2.1. Participants and Procedures

Sample size estimation was performed using SPSS 26.0 software. Based on previous research data, the population standard deviation (σ) of perceived social support was set at 10.79 (Jia et al., 2022). With a confidence level (μ) of 0.95, a significance level (α) of 0.05, and a margin of error (δ) of 0.8, the initial sample size was calculated as *n* = (μ_α_^σ^/δ)^2^. Considering potential non-response or invalid responses with an estimated response rate of 90%, the minimum required sample size was determined to be 776 participants. Furthermore, given the application of Latent Transition Analysis in this study, a sample size between 300 and 1000 was deemed necessary to ensure model robustness and result reliability (Nylund-Gibson et al., 2023).

Questionnaires were administered across four middle schools in H Province, China. The study protocol was approved by the Scientific Ethics Committee (Ethics Review Number: KJ2024-128-01). Prior to data collection, researchers obtained administrative approval by presenting the study objectives and procedures to school administrators and faculty members while simultaneously distributing informed consent forms to potential participants. During on-site data collection, the researchers reiterated the study purpose and procedures in detail. All participants provided written informed consent under conditions of full disclosure and voluntary participation.

The first wave of the survey was conducted in September 2024 (timepoint of the first measurement, hereinafter T1), with 1185 questionnaires distributed. After excluding incomplete and unreliable responses, 1145 valid questionnaires were obtained, yielding a valid response rate of 96.62%. The second wave of the survey took place in March 2025 (timepoint of the second measurement, hereinafter T2), with a six-month interval between measurements; due to reasons such as student transfers and relocations, some students being unable to participate in the second survey, as well as incomplete or unreliable responses, the final analytical sample comprised 995 students with valid data at both time points. The two measurement periods coincided with distinct phases of interpersonal relationship development–the “formation phase” at the beginning of the academic year and the “consolidation-deepening phase” six months later. This temporal design provided an ideal ecological context for examining transitions in perceived social support categories, as the six-month interval was sufficient for meaningful changes to occur while minimizing attrition. The number of participants lost to attrition was 150 (number of attrited participants = number of valid participants at T1—number of participants with valid data at both time points). The participant attrition rate was 13.1% (attrition rate = number of attrited participants/number of valid questionnaires at T1 × 100%), which is within an acceptable range. The sample included 487 males (48.9%) and 508 females (51.1%), as well as 555 students from urban areas (55.8%) and 440 from rural areas (44.2%).

### 2.2. Measures

#### 2.2.1. The Multidimensional Scale of Perceived Social Support

This scale was developed by Zimet et al. and revised by Yan Biao-bin et al. and consists of three subscales: Perceived Family Support, Perceived Peer Support, and Perceived Teacher Support (Ye & Zhu, 2022). Each scale consists of four items with a 7-point scale (1 = strongly disagree, 7 = strongly agree). For subscale scores, mean values below 3 indicate low levels of perceived social support, scores between 3 and 5 reflect moderate levels, and scores above 5 indicate high levels. The Cronbach’s α values for the full scale were 0.91 at T1 (timepoint of the first measurement, hereinafter T1) and 0.94 at T2 (timepoint of the second measurement, hereinafter T2). For the subscales, the Cronbach’s α values were 0.89 (family), 0.89 (peers), and 0.93 (teachers) at T1 and 0.89 (family), 0.89 (peers), and 0.94 (teachers) at T2.

#### 2.2.2. Delaware Social and Emotional Competency Scale

The scale was originally developed by George Bear and Chunyan Yang and subsequently translated and culturally adapted by Xinxin Zhu, with its conceptual framework grounded in the core social–emotional competencies framework established by CASEL (Yu et al., 2025). The scale is divided into four dimensions: Social awareness, Self-management, Peer relationships, and Responsible decision-making. Social awareness refers to the ability to accept multiple perspectives and demonstrate empathy; self-management refers to the ability to successfully regulate emotions, cope with stress, control impulses, and motivate oneself; peer relations is the ability to establish and maintain positive relationships with diverse people; and responsible decision-making involves assessing the consequences of behaviors and considering the well-being of the individual and others (H. Song & Zhou, 2021). Given the ethical concerns in the testing process and the inappropriateness of self-awareness-related items for middle school students, George Bear and Chunyan Yang excluded the self-awareness dimension of the CASEL framework from the measurement scope of this scale (Chu, 2024). This questionnaire contains 12 items and is scored on a 4-point Likert scale (1 = not at all like me, 4 = very much like me). The Cronbach’s α values for this scale were 0.78 and 0.79 for the T1 and T2 tests, respectively.

### 2.3. Data Processing and Analysis

In the first step, SPSS 26.0 was used to enter the data; Latent Profile Analysis and Latent Transition Analysis were conducted using Mplus 8.0, with the three dimensions of perceived social support (perceived family support, perceived peer support, and perceived teacher support) as indicators to establish the latent profile model. Following the principle of iterative comparison, starting from a single-class baseline model, the number of classes was incrementally increased. Model fit was evaluated based on specific criteria until the fit indices reached their optimal level (Bauer, 2022), the specific fit indices included: (1) the relative fit index, including AIC, BIC, and aBIC, where the lower the value, the better the model fit; (2) entropy, where the closer the Entropy value is to 1, the more accurate the classification; (3) LMRT and BLRT, which indicate the difference between two neighboring models; if the results of LMRT and BLRT are significant, then the Kth model is better than the K-1st model; and (4) a category probability in each subgroup of no less than 5% (Mathew & Doorenbos, 2022). In the second step, a latent transition model was established, and transition probabilities were used to characterize the transition in adolescents’ perceived social support categories between the two time points. In the third step, multinomial logistic regression analyses and hierarchical linear regression analyses were used to explore the influences of adolescents’ transitions in their perceived social support categories, as well as the effects of category transition patterns on their social–emotional competence.

## 3. Results

### 3.1. Common Method Bias Test

All measures of perceived social support and social–emotional competence were subjected to unrotated exploratory factor analysis. Seven and eight common factors with eigenvalues greater than 1 were extracted from T1 and T2, respectively. The total variance explained by the first factor was 24.13% at T1 and 30.52% at T2. These values were less than the 40% critical value, indicating that no significant common method bias existed in this study.

### 3.2. Determining the Number of Latent Classes of Adolescents’ Perceived Social Support

Latent Profile Analysis was conducted to identify adolescents’ perceived social support classes. The model fit indices for the LPA at T1 and T2 are presented in Table 1. As the number of classes increased, the relative fit indices at both time points demonstrated a decreasing trend, with the rate of decline gradually stabilizing beyond the four-class model, indicating progressive improvement in the model fit. Concurrently, Entropy values systematically increased with additional classes, with the Entropy values for the four-class model all over 0.8 and the probability of each category being greater than 5%. However, when classified into five categories, we observed a situation in which the percentage of samples in a category was less than 5%, indicating that the fit of the five-class model did not meet the criteria for potential profile analysis. Therefore, the four-class model for adolescents’ perceived social support was determined to be the best model.

The quality of the four-class perceived social support model was examined using average attribution probabilities, which reflect the precision of latent profile classification. As shown in Table 2, the probabilities of accurate classification among the four classes at T1 and T2 were 92–96% and 89–92%, respectively, indicating that the four-category model results are credible.

**Figure 1:**
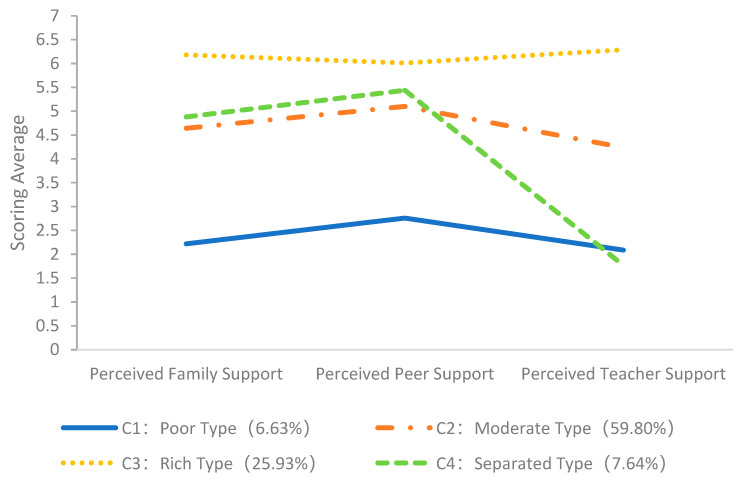
Mean scores for the four latent classes of T1 adolescent perceived social support.

**Figure 2:**
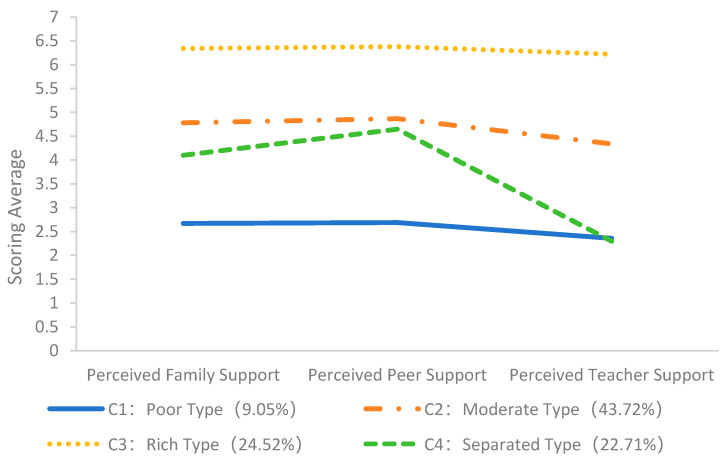
Mean scores for the four latent classes of T2 adolescent perceived social support.

The four classes of perceived social support were named based on adolescents’ average scores across three dimensions. Figure 1 and Figure 2 display the mean score of the four classes at T1 and T2, respectively. C1 exhibited mean scores between 1 and 3 on the dimensions of perceived social support (mean scores across the three dimensions were 2.09–2.76 at T1 and 2.36–2.69 at T2), which were consistently lower than the other three classes. Therefore, this low-level combination of resource accumulation was named the Poor type. C2 exhibited intermediate-level scores on the dimensions of perceived social support (mean scores across the three dimensions were 4.24–5.10 at T1 and 4.34–4.87 at T2), with scores ranging from 4 to slightly above 5. Therefore, it was named the Moderate type. C3 showed mean scores between 5 and 7 on the dimensions of perceived social support (mean scores across the three dimensions were 6.01–6.29 at T1 and 6.22–6.38 at T2), which were consistently higher than the other three classes. Therefore, this high-level combination of resource accumulation was named the Rich type. C4 exhibited a high mean score on the dimension of perceived peer support (5.44 at T1, 4.65 at T2) but a low mean score on the dimension of perceived teacher support (1.77 at T1, 2.30 at T2); overall perceived social support presented a disconnected pattern, and it was named the Separated type.

To explore whether the classes of adolescent perceived social support are heterogeneous, we conducted a one-way ANOVA using latent classes as the independent variables and the three perceived social support dimensions (family, peer, teacher) as dependent variables. The results are presented in Table 3; significant differences were found among the four adolescent classes on all three dimensions of perceived social support. Furthermore, the results of post-hoc multiple comparisons showed that most indicators had significant pairwise differences between classes, indicating that the latent classification is valid.

### 3.3. Analysis of Potential Transitions in Adolescents’ Perceived Social Support

Latent Transition Analysis was used to explore the dynamic developmental process of adolescent perceived social support classes. The probabilities for adolescents to either remain in their original class (latent status probabilities) or transition to other classes (latent transition probabilities) are shown in Table 4. Overall, compared with the other three classes, the Poor type had the lowest stability in the transition process. Specifically, from T1 to T2, the probability of the Poor type remaining in the same group was 27%, with 28% of individuals transitioning to the Separated type and a larger proportion (44%) transitioning to the Moderate type. The probability that the Moderate type would remain in the original group was 38%, with only 8% transitioning to the Poor type, 21% to the Rich type, and 33% to the Separated type. The probability that the Rich type would remain in the original group was 38%, with 10% transitioning to the Poor type, 36% transitioning to the Moderate type, and 16% transitioning to the Separated type. The probability that the Separated type would remain in the original group was 34%, with only 3% of individuals transitioning to the Poor type, 59% to the Moderate type, and 4% to the Rich type.

### 3.4. Factors Influencing the Transition in Potential Categories of Adolescents’ Perceived Social Support

Table 5 presents the transition patterns of adolescents’ perceived social support latent classes. Specifically, “1→1” represents individuals who remained as the Poor type at both T1 and T2; “1→2” denotes those who transitioned from the Poor type at T1 to the Moderate type at T2; “1→3” indicates a transition from the Poor type at T1 to the Rich type at T2; and “1→4” reflects a transition from the Poor type at T1 to the Separated type at T2. By analogy, we observed a total of 16 transition patterns for adolescents’ perceived social support from T1 to T2.

To further investigate the factors influencing transitions between adolescents’ perceived social support classes, multinomial logistic regression analyses were conducted. Taking the participants who remained in the same group during the transition patterns as the reference group, this study examined the effects of gender, age, and boarding status on adolescents’ transition patterns in perceived social support. As shown in Table 6, when those who remained in the Poor type were used as the reference, age increased the probability of the Poor type transitioning to the Moderate (OR = 2.16, 95%CI: 1.20~3.88) or Rich types (OR = 2.31, 95%CI: 1.02~5.25). When those who remained in the Moderate type were used as the reference, boarding increased the probability that those in the Moderate type would transition to the Poor type (OR = 2.84, 95%CI: 1.43~5.63). When using those who remained in the Rich type as a reference, males had a decreased probability of transitioning from the Rich type to the Separated type (OR = 0.40, 95%CI: 0.19~0.84). When those who remained in the Separated type were used as a reference, age increased the probability that the Separated type would transition to the Moderate (OR = 1.74, 95%CI: 1.04~2.93) or Rich types (OR = 2.06, 95%CI: 1.03~4.12), and boarding reduced the probability that those in the Separated type would transition to the Moderate type (OR = 0.25, 95%CI: 0.08~0.81).

### 3.5. Impact of Potential Transition Patterns in Adolescents’ Perceived Social Support on Social–Emotional Competence

To examine the effect of adolescent perceived social support transition patterns on social–emotional competence, a hierarchical linear regression analysis was conducted. The transition patterns of perceived social support were treated as the independent variable and were dummy-coded; social–emotional competence and its dimensions at T2 were used as the dependent variables, with gender, age, and boarding status serving as control variables. Due to space constraints, only the significant transition patterns are reported here (Table 7). When the constant was the pattern that remained in the Poor type, the transition patterns from Poor, Moderate, and Separated to Rich, as well as the transition pattern that remained in the Rich type, positively predicted social–emotional competence at T2. The transition from Moderate to Separated, as well as remaining in the Rich and Separated types, positively predicted the responsible decision-making dimension at T2. The transition pattern that remained in the Rich type positively predicted the social awareness and self-management dimensions at T2. The transition patterns from Poor, Moderate, and Separated to Rich; remaining in the Rich type; remaining in the Moderate type; and transitioning from Poor to Moderate positively predicted peer relationships at T2 (all *p* < 0.05).

## 4. Discussion

### 4.1. Potential Profile Results of Adolescent Perceived Social Support

This present study explored four types of adolescents’ predicted social support: Poor, Moderate, Rich, and Separated, supporting H1. Previous scholars have used structural magnetic resonance imaging, behavioral cognitive testing, and genetic/methylomic data from adolescent longitudinal cohorts to determine the heterogeneity in the developmental patterns of whole-brain gray matter volume (GMV) among adolescents. GMV, a key neural substrate for cognitive development, largely influences individuals’ ability to process and utilize environmental resources (R. Shi et al., 2024). This finding provides a robust neurobiological basis for the Cumulative Ecological Resources Theory, indicating that differences in physiological development among individuals often directly influence their ability to perceive surrounding ecological resources, which, in turn, leads to the differentiation of perceived social support categories. Notably, a previous study identified four types of perceived social support: extremely low, low, medium, and high, the difference between the classification results and our study may arise from differences in sample size (Mai et al., 2021).

At T1 and T2, the proportion of adolescents in the moderate and rich types exceeded 65% of the total sample, indicating that most adolescents had a moderate to high level of perceived social support. These adolescents were able to obtain instrumental and emotional support from their social environment, which made them more confident and proactive in their social interactions, in line with previous research findings (H. Wang et al., 2023). Special attention should be paid to the Poor and Separated types. Adolescents in the Poor type often experience a certain degree of detachment from social relationships. Fortunately, the proportion of adolescents classified as Poor type was the lowest (6.63% at T1 and 9.05% at T2). In educational practice, it is necessary to construct a system based on collaborative education among schools, families, and society to expand the types and quantity of ecological resources for adolescents, thereby comprehensively enhancing the overall level of perceived social support. For adolescents in the Separated type, given the extensive and uneven distribution of ecological resources, those in resource-scarce contexts tended to present more pronounced dependence on their peers, as peer resources are relatively easier to obtain (Zhong & Ni, 2021). Moreover, the Significant Other Theory indicates that during adolescence, an individual’s social network gradually shifts from attachment to parents and teachers to attachment to peers. As a result, adolescents in the Separated category tend to perceive a high level of peer support (Xiang et al., 2024). Meanwhile, the development of self-awareness and psychological resources during adolescence is typically accompanied by a corresponding decrease in the need for teacher guidance. Under the situational influence of new teacher–student interaction patterns, teachers may choose to adopt non-directive or guiding support methods, which can lead to adolescents perceiving a loss of teacher support resources. However, previous research has shown that the quality of teacher-student interactions is a significant predictor of adolescent peer relationships (Endedijk et al., 2022). Therefore, leveraging the added value of perceived peer support resources and exploring how to effectively extend this effect to the realm of teacher support has become a key focus for interventions targeting separated adolescents.

### 4.2. Outcomes of Latent Transition in Adolescents’ Perceived Social Support

The different classes of perceived social support exhibited distinct patterns of stability and transition, which supports H2. Specifically, the Poor type exhibited greater category mobility and tended to move more often towards the Moderate type, possibly because some adolescents, in the early stage of the survey, presented a “helpless” characteristic in the acquisition of ecological resources due to environmental changes and adaptation difficulties when entering a new school. However, as time passed and the adolescents’ new environment began to reveal positive features such as safety, order, and harmonious interpersonal relationships, the adolescents’ ecological resource development showed a “Gain Spiral Effect” (M. Shi et al., 2023). This effect promotes the reintegration of ecological resources, a process which in turn generates further resource gains, ultimately, this guides adolescents’ perceived social support onto a more positive developmental trajectory (Duan et al., 2024). This pattern of developmental transition reveals how certain individuals transition from a state of relatively limited perceived social support to a state of abundant perceived social support (Huang et al., 2024). Those in the Moderate and Rich types exhibited strong stability. However, some adolescents transitioned from the medium-high perceived social support group to the lower-level group. The Cumulative Ecological Resource “Loss Spiral Model” suggests that obstructive stressors, by inducing negative psychological states such as anxiety and depression, trigger a “learned helplessness” response among individuals regarding resource loss. This emotional state diminishes adolescents’ ability to actively seek and perceive support from others, thereby exacerbating the vicious cycle of resource depletion (Ford et al., 2023). Empirical studies also indicate that adolescents’ depression and anxiety can impair their ability to perceive social support and exert a negative predictive effect on perceived social support (D. D. Shi et al., 2022). Compared to the acquisition of ecological resources, the loss of such resources exerts a more profound and enduring impact on individuals. Therefore, in educational practice, greater attention should be actively directed toward adolescents experiencing a decline in perceived social support.

The Poor, Moderate, and Rich types all contained some individuals who transitioned to the Separated type. From the Theory Perspective of Ecological Resource Compensation Effects, this phenomenon may stem from the differential perception of contextual support changes among highly environmentally sensitive adolescents. When teachers fail to provide adequate individualized support, this group becomes more susceptible to perceiving the risk of losing teacher support resources, consequently leading to a significant decline in their perceived level of teacher support (Tang et al., 2024; Zeng et al., 2022). In contrast, the more readily available perceived peer support gradually becomes a crucial compensatory resource for adolescents’ physical and mental development, offsetting the loss of teacher support resources; this phenomenon ultimately leads to a structural separation in the sources of perceived social support (De Beer et al., 2024). H. Wang et al. (2023) further confirmed this tendency of separation through category research on perceived social support. Separated-type adolescents showed a greater tendency to transition to the Moderate type, reflecting a transition from an imbalanced to a balanced state in perceived social support resources. Social Adaptation Theory posits that individuals interact with their social environment to achieve harmony and stability. This adaptive process may facilitate adolescents’ transition from psychological imbalance to equilibrium, thereby enhancing their utilization of social support resources. These findings further support the cumulative and integrative effects of ecological resources—as adolescents develop, resources within their ecosystems (e.g., family, school, community) progressively accumulate and integrate (Tian et al., 2024). Further research pointed out that a balanced integration of perceived social support resources is key to adolescents’ resource attainment (Juneja et al., 2024).

### 4.3. Gender, Age, and Boarding Status Significantly Predict Latent Class Transitions in Adolescents’ Perceived Social Support

Gender is a factor influencing the dynamic changes in adolescents’ perceived social support profiles. Males are less likely to shift from the Rich to the Separated type, indicating that females are more likely to exhibit low teacher and high peer support levels during adolescence. X. Zhao’s (2022) study on the mental health of high school students in China, Japan, and South Korea also showed that boys have significantly higher perceived teacher support than girls. This is possibly because males present higher levels of proactive personality traits and are more willing to participate in class activities, leading them to perceive superior teacher support resources (Q. Guo, 2024). Conversely, females tend to seek enthusiastic and dependent peer relationships as their gender roles develop, resulting in higher perceived peer support (Y. L. Wang et al., 2024). Age can also enhance adolescents’ levels of perceived social support in longitudinal time dimensions and tends to be balanced between family, teachers, and peers, which is consistent with previous research (J. Z. Li et al., 2024). The Future Time Insight perspective indicates that adolescents’ future time insight improves with age and serves as a protective factor for perceived social support development (C. M. Li, 2022). Boarding students, due to having less interaction with family members, experience fewer intimate emotional connections and have limited autonomy, making it difficult for them to engage with a broader peer group and lowering their perceived social support. Compared to non-boarding students, boarding students are more likely to remain within the Separated group. This separation may occur because peer support is particularly prominent among boarding students, while their long-term separation from family leads to higher expectations of teacher support. When these expectations are unmet, these students may perceive a lack of teacher support. Therefore, in educational practice, teachers should provide greater assistance to boarding students and help them enhance their self-management and self-care abilities.

### 4.4. The Impact of Adolescents’ Perceived Social Support Classes Transition Patterns on Social–Emotional Competence

The transition patterns from the other three types to the Rich type of perceived social support, as well as the stability in maintaining the Rich type, demonstrated positive predictive effects on social–emotional competence and its subdomains at T2. This indicates that when adolescents successfully build a perceived social support profile characterized by high resource accumulation, this positive transition process promotes the development of their social–emotional competence; therefore, these findings validate H3. The “Contextual Breadth Effect” of cumulative ecological resources posits that the synergistic accumulation of cross-contextual resources (family, school, and peers) exhibits significantly stronger predictive validity for adolescent developmental outcomes compared to that of single-context resources (S. Y. Yang et al., 2023). When adolescents’ perceived social support transitions to or remains within the Rich type, there is a significant increase in the types and quantities of support resources across multiple contexts, including family, peers, and teachers. Compared to individual contexts, this multilinear development trend of resources in multiple contexts has a substantial positive impact on the development of adolescents’ social–emotional competence. Previous researchers have also noted that in the ranking of contextual contributions to the development of individual social–emotional competence, family, peers, and teacher support respectively account for 29.22%, 21.39%, and 0.15% of the variance in social–emotional competence, and these three factors together explain the development of social–emotional competence (S. Z. Chen, 2023). The Cumulative Ecological Resources Theory further proposes the “Vertical Accumulation Effect” of resources; this effect means that the ecological resources an adolescent acquires at any given time point can influence positive developmental outcomes, not only in the present but also in the future, through the mediating role of future time perspective (Jin et al., 2022). That is to say, in the process of adolescents’ perceived social support transitioning to the Rich type from the other three types, the promotion and protection effects of perceived social support resources are extended to positive social–emotional development at the next time point. Previous large-scale longitudinal research by Tsai et al. (2023) has similarly demonstrated significant longitudinal associations between the accumulation of psychological resources and positive psychosocial development during adolescence.

Notably, the transition from the Moderate type to the Separated type, as well as the maintenance of the Separated type, can positively predict adolescents’ responsible decision-making levels. In these two transition patterns, adolescents’ perceived social support from peers remains consistently high. The dual mechanism of the peer system indicates that peers can more rapidly share information resources, which helps adolescents integrate multidimensional contextual cues and thus construct a more complete cognitive representation for decision-making. Moreover, the acquisition of peer role models can help adolescents adopt responsible approaches when facing decisions through observational learning and vicarious reinforcement (W. Zhang et al., 2023). Experimental research has further demonstrated that when adolescents complete tasks under the influence of peer relationships, compared to completing tasks alone, there is greater activation in the amygdala, a brain region responsible for risk assessment; this suggests that peer presence and the social observation of peers may increase the likelihood of adolescents making rational decisions (Van Duijvenvoorde et al., 2022). This study also revealed that the number of patterns positively predicting the peer relationship dimension was highest among the 16 transition patterns. These transition patterns all exhibited an increase in the level of perceived social support or maintenance at a medium-to-high level. This result indicates that the peer relationship dimension is highly sensitive to the transition patterns of perceived social support. Empirical research further demonstrated that the quality of peer friendships provides an important support resource in the psychological socialization process of adolescents and has a significant predictive effect on the development of perceived social support (S. Li & Zhang, 2024).

### 4.5. Limitations and Future Research

This study innovatively examined the present issue from the Theory Perspective of Cumulative Ecological Resources Theory, revealing the potential categories of adolescents’ perceived social support and the impact of category transition patterns on their social–emotional competence. This study not only extends and supplements existing theories regarding perceived social support and social–emotional competence but also provides a new theoretical perspective for understanding the protective factors in the development of individual social–emotional competence. Additionally, this study offers certain references to improve the circumstances of adolescents in the four categories of perceived social support and formulate intervention measures, as well as a new perspective that could enhance adolescents’ social–emotional competence. However, this study also has some limitations. Firstly, the research on the psychological mechanisms and adaptive performance of Separated adolescents is not sufficiently comprehensive. Future research should focus on exploring the unique mechanisms of separated adolescents in the process of psychological development and how these mechanisms affect their adaptive performance. Secondly, the present data come from adolescents’ self-reports, which are prone to both self-reporting and recall bias. In the future, research could include parents’ and teachers’ reports and adopt a triangulated multi-subject reporting model to comprehensively explore adolescents’ perceived social support status, in order to enhance the authenticity and reliability of the results. Finally, the duration of this study was relatively short, which hindered our ability to more deeply explore the potential transformation process of adolescents’ perceived social support. Future studies could also consider a longer duration of follow-up to further reveal the transformation process of adolescents’ perceived social support.

## 5. Conclusions

Based on the Theory Perspective of Cumulative Ecological Resources, this study successfully examined the effect of developmental characteristics of adolescents’ perceived social support on social–emotional competence. The study found that adolescents’ perceived social support exhibits qualitative heterogeneity (RQ1). Specifically, a conclusion that not only confirmed the hypothesis of at least three distinct profiles of perceived social support but also further identified four more precise profiles: Poor, Moderate, Rich, and Separated. The Poor type showed the lowest levels across all three dimensions, the Moderate type demonstrated intermediate support levels in all dimensions, the Rich type exhibited the highest support levels consistently, and the Separated type was characterized by coexisting high perceived peer support but low perceived teacher support. This finding addresses a key limitation in prior research by shifting the focus from the quantity of support to the qualitative configurations of resources across contexts. The study further revealed that the different categories of adolescents’ perceived social support exhibit both stable and transitional pathways over time (RQ2), transcending the static perspective of previous cross-sectional studies and uncovering the non-linear characteristics of the development of adolescents’ perceived social support from a longitudinal and dynamic perspective, which supported Hypothesis 2. Specifically, the Poor type showed higher transition probabilities, whereas the Moderate and Rich types demonstrated higher stability. Some adolescents in the Poor, Moderate, and Rich types transitioned to the Separated type. Those of the Separated type were found to have a higher probability of transitioning to the Moderate type. Gender, age, and boarding status significantly influenced these class transitions. Finally, this study’s key contribution is longitudinally linking transitions in perceived social support profiles to social–emotional competence. Specifically, transitioning to the Rich type from the other three types, as well as remaining in the Rich type, was found to positively predict social–emotional competence at T2 (RQ3). This conclusion suggests that in educational practice, the focus of interventions should shift from static resource stock to dynamic resource accumulation—that is, helping adolescents learn to maintain support rather than just receive temporary aid. It also provides a new perspective for enhancing adolescents’ social–emotional competence.

## Figures and Tables

**Table 1:** Fit indices of the LPA models for adolescents’ perceived social support at T1 and T2 (*n* = 995).

Time	Model	*AIC*	*BIC*	*aBIC*	*Entropy*	*LMR(p)*	*BLRT(p)*	Categorical Probability
T1	1	10,272.83	10,302.25	10,283.19	-	-	-	1.00
	2	9863.56	9912.58	9880.82	0.82	0.036	<0.001	0.12/0.88
	3	9480.91	9549.55	9505.09	0.83	<0.001	<0.001	0.66/0.06/0.27
	**4**	**9302.75**	**9391.01**	**9333.83**	**0.91**	**<0.001**	**<0.001**	**0.07/0.60/0.26/0.08**
	5	9247.02	9354.88	9285.01	0.92	<0.001	<0.001	0.07/0.01/0.60/ 0.26/0.08
T2	1	10,373.90	10,403.32	10,384.26	-	-	-	1.00
	2	9444.11	9493.14	9461.38	0.84	<0.001	<0.001	0.69/0.31
	3	9020.06	9088.70	9044.24	0.89	<0.001	<0.001	0.10/0.61/0.28
	**4**	**8850.27**	**8938.52**	**8881.35**	**0.89**	**<0.001**	**<0.001**	**0.09/0.44/0.25/0.23**
	5	8793.47	8901.33	8831.46	0.89	0.100	<0.001	0.21/0.09/0.02/ 0.43/0.25

Note: Bold indicates the best-fit model and the case of the fit indices.

**Table 2:** Average attribution probabilities of latent classes at T1 and T2 (*n* = 995).

Time	Categories	Number of Subjects	Percentage	Probability of Attribution			
				C1	C2	C3	C4
T1	C1	66	6.63%	0.96	0.01	0.00	0.03
	C2	595	59.80%	0.00	0.96	0.02	0.01
	C3	258	25.93%	0.00	0.05	0.95	0.00
	C4	76	7.64%	0.03	0.05	0.00	0.92
T2	C1	90	9.05%	0.89	0.02	0.00	0.10
	C2	435	43.72%	0.00	0.95	0.02	0.03
	C3	244	24.52%	0.00	0.03	0.97	0.00
	C4	226	22.71%	0.04	0.05	0.00	0.92

Note: C1 = Class1, C2 = Class2, C3 = Class3, C4 = Class4.

**Table 3:** Class differences in perceived social support dimensions among adolescent latent classes (*M* ± *SD*).

Time	Dimensions	Latent Classes of Perceived Social Support				*F-Test*	$\eta_{p}^{2}$	*Post-Hoc* Tests
		C1	C2	C3	C4			
T1	Perceived Family Support	2.22 ± 0.81	4.64 ± 1.01	6.18 ± 0.86	4.88 ± 0.95	342.509 ***	0.51	C1 < C2 < C3, C3 > C4, C1 < C4
	Perceived Peer Support	2.76 ± 0.93	5.10 ± 0.99	6.01 ± 0.94	5.44 ± 0.70	209.827 ***	0.39	C3 > C4 > C2 > C1
	Perceive Teacher Support	2.09 ± 0.82	4.24 ± 0.59	6.29 ± 0.64	1.77 ± 0.52	1593.504 ***	0.83	C3 > C2 > C1 > C4
T2	Perceived Family Support	2.67 ± 0.92	4.78 ± 0.84	6.34 ± 0.67	4.10 ± 0.85	553.496 ***	0.63	C3 > C2 > C4 > C1
	Perceived Peer Support	2.69 ± 0.65	4.87 ± 0.74	6.38 ± 0.59	4.65 ± 0.86	611.845 ***	0.65	C3 > C2 > C4 > C1
	Perceived Teacher Support	2.36 ± 0.83	4.34 ± 0.54	6.22 ± 0.71	2.30 ± 0.49	1931.024 ***	0.85	C3 > C2 > C1, C2 > C4, C3 > C4

Note: C1 = Poor Type, C2 = Moderate Type, C3 = Rich Type, C4 = Separated Type. *** *p* < 0.001.

**Table 4:** Potential state probabilities and potential transition probabilities for T1–T2 (*n* = 995).

	C1	C2	C3	C4
latent state probability				
T1	0.07	0.60	0.26	0.08
T2	0.09	0.44	0.25	0.23
T1 → T2 transition probabilities				
1	0.27	0.44	0.01	0.28
2	0.08	0.38	0.21	0.33
3	0.10	0.36	0.38	0.16
4	0.03	0.59	0.04	0.34

**Table 5:** Adolescents appreciate the different patterns of transitions in the type of social support from T1→T2.

Cluster	Poor Maintained Group	Poor Transformation Group			Moderate Maintained Group	Moderate Transformation Group		
transition patterns	1→1	1→2	1→3	1→4	2→2	2→1	2→3	2→4
cluster	Rich Maintained Group	Rich Transformation Group			Separated Maintained Group	Separated Transformation Group		
transition patterns	3→3	3→1	3→2	3→4	4→4	4→1	4→2	4→3

**Table 6:** Occurrence ratio of transition probabilities under the influence of demographic variables.

Latent State	Factor	C1			C2			C3			C4		
		*B (SE)*	*OR*	*95%CI*	*B (SE)*	*OR*	*95%CI*	*B (SE)*	*OR*	*95%CI*	*B (SE)*	*OR*	*95%CI*
T1–T2													
C1	gender ^a^	-	-	-	0.72(0.75)	2.05	[0.47,8.84]	1.29(0.96)	3.63	[0.55,23.93]	−0.38(0.86)	0.69	[0.13,3.73]
	age	-	-	-	0.77 **(0.30)	2.16	[1.20,33.88]	0.84 *(0.42)	2.31	[1.02,5.25]	0.38(0.31)	1.46	[0.80,2.67]
	boarder ^b^	-	-	-	−0.34(0.69)	0.71	[0.18,2.76]	0.04(0.94)	1.04	[0.17,6.52]	−0.28(0.74)	0.75	[0.18,3.21]
C2	gender	0.44(0.31)	1.56	[0.86,2.83]	-	-	-	0.28(0.23)	0.84	[0.22,1.33]	0.27(0.21)	1.31	[0.88,1.96]
	age	−0.22(0.12)	0.80	[0.63,1.02]	-	-	-	0.06(0.09)	1.06	[0.89,1.27]	0.01(0.08)	1.01	[0.86,1.19]
	boarder	1.04 **(0.35)	2.84	[1.43,5.63]	-	-	-	0.32(0.24)	1.38	[0.87,2.19]	0.21(0.21)	1.23	[0.82,1.85]
C3	gender	−0.76(0.57)	0.47	[0.15,1.44]	−0.33(0.29)	0.72	[0.41,1.27]	-	-	-	−0.92 *(0.38)	0.40	[0.19,0.84]
	age	−0.11(0.23)	0.90	[0.58,1.40]	0.16(0.11)	1.18	[0.95,1.46]	-	-	-	0.20(0.14)	1.22	[0.92,1.61]
	boarder	0.43(0.60)	1.53	[0.47,4.96]	−0.10(0.30)	0.91	[0.51,1.63]	-	-	-	−0.34(0.39)	0.71	[0.33,1.54]
C4	gender	0.50(0.91)	1.64	[0.28,9.81]	0.17(0.60)	1.18	[0.37,3.79]	−0.68(0.978)	0.51	[0.11,2.31]	-	-	-
	age	−0.17(0.40)	0.84	[0.38,1.85]	0.55 *(0.27)	1.74	[1.04,2.93]	0.72 *(0.35)	2.06	[1.03,4.12]	-	-	-
	boarder	1.15(1.19)	3.16	[0.31,32.48]	−1.38 *(0.59)	0.25	[0.08,0.81]	−0.46(0.70)	0.63	[0.16,2.48]	-	-	-

Note: C1 = Poor type, C2 = Moderate type, C3 = Rich type, C4 = Separated type. ^a^: female students in the reference group, ^b^: non-boarding students in the reference group; * *p* < 0.05, ** *p* < 0.01.

**Table 7:** Stratified linear regression analysis of the effects of adolescents’ perceived social support transition patterns on social–emotional competence and dimensions.

Constants and Independent Variables	Pathway	Model 1			Model 2		
		Social–Emotional Competence					
		*β*	*t*	*p*	*β*	*t*	*p*
constant			18.70	**<0.001**		16.20	**<0.001**
gender		0.01	0.10	0.917	0.03	0.96	0.337
age		0.01	0.08	0.937	0.01	0.09	0.930
boarder		0.02	0.47	0.642	−0.01	−0.05	0.961
paradigm shift	1→3	-	-	-	0.10	2.67	**0.008**
	2→3	-	-	-	0.24	3.23	**0.001**
	3→3	-	-	-	0.36	4.62	**<0.001**
	4→3	-	-	-	0.12	3.02	**0.002**
		Responsible Decision-making					
constant			14.73	**<0.001**		13.12	**<0.001**
gender		−0.02	−0.65	0.519	−0.01	−0.20	0.842
age		0.04	1.11	0.265	0.04	1.27	0.205
boarder		0.05	1.65	0.100	0.04	1.36	0.173
paradigm shift	2→4	-	-	-	0.20	2.37	**0.018**
	3→3	-	-	-	0.19	2.40	**0.017**
	4→4	-	-	-	0.10	2.13	**0.034**
		Social awareness					
constant			12.10	**<0.001**		10.09	**<0.001**
gender		0.13	4.06	**<0.001**	0.15	4.73	**<0.001**
age		0.07	2.10	0.036	0.07	2.35	0.036
boarder		0.05	1.65	0.100	0.04	1.42	0.100
paradigm shift	3→3	-	-	-	0.17	2.14	0.036
		Self-management					
constant			13.95	**<0.001**		12.07	**<0.001**
gender		0.00	0.03	0.974	0.03	0.88	0.377
age		−0.01	−0.18	0.860	0.00	−0.02	0.984
boarder		0.03	1.04	0.300	0.02	0.60	0.551
paradigm shift	3→3	-	-	-	0.23	2.82	**0.005**
		Peer Relationship					
constant			14.16	**<0.001**		12.10	**<0.001**
gender		−0.02	−0.62	0.533	−0.01	−0.17	0.868
age		−0.04	−1.18	0.237	−0.05	−1.52	0.129
boarder		−0.04	−1.36	0.175	−0.05	−1.81	0.070
paradigm shift	1→2	-	-	-	0.13	2.79	**0.005**
	1→3	-	-	-	0.12	3.47	**0.001**
	2→2	-	-	-	0.26	2.50	**0.013**
	2→3	-	-	-	0.28	3.90	**<0.001**
	3→3	-	-	-	0.38	4.93	**<0.001**
	4→3	-	-	-	0.14	3.48	**0.001**

Note: Boldface indicates that the results are statistically significant (*p* < 0.05).

## Data Availability

Data will be made available upon request.

## Abbreviations

The following abbreviations are used in this manuscript:

SEC	social–emotional competence
CASEL	Collaborative for Academic, Social, and Emotional Learning
PSS	perceived social support
LPA	Latent Profile Analysis
LTA	Latent Transition Analysis
